# The relationship between non-HDL cholesterol to HDL cholesterol ratio (NHHR) and anemia: A cross-sectional study of NHANES, 2009 to 2016

**DOI:** 10.1097/MD.0000000000040976

**Published:** 2024-12-13

**Authors:** Nengneng Cao, Jiajia Wang, Jinli Zhu, Xunyi Jiao, Furun An, Zhimin Zhai

**Affiliations:** a Department of Hematology, The Second Affiliated Hospital of Anhui Medical University, Hefei, Anhui, China; b Department of Hematology, Tongling People’s Hospital, Tongling, Anhui, China.

**Keywords:** anemia, cross-sectional study, NHANES, NHHR

## Abstract

The non-HDL cholesterol to HDL cholesterol ratio (NHHR) is a newly developed metric that represents the ratio of non-HDL cholesterol to HDL cholesterol. Anemia is a prevalent public health concern affecting all age groups. Our purpose is to investigate the connection between NHHR and the prevalence of anemia, as well as to explore their potential interactions. A total of 17,019 participants were incorporated into this research. NHHR was calculated as the scale of non-HDL cholesterol to HDL cholesterol. According to WHO criteria, anemia at sea level is diagnosed with a hemoglobin level (g/dL) of <12 for women and <13 for men. This study utilized multivariate linear regression, threshold effect analysis, smooth curve fitting, multivariate logistic regression as well as subgroup analysis, to investigate the linkage between the NHHR and anemia. After complete adjustment, the model indicated a strong inverse relationship between NHHR and the prevalence of anemia (the odds ratio [OR] was 0.82 with a 95% confidence interval of 0.78–0.86), suggesting that an incremental increase in NHHR correlates with an 18% decrease in the prevalence of anemia. Segmenting NHHR into quartiles maintained this significant association. The prevalence of anemia was 51% lower among participants in the highest NHHR quartile compared to those in the lowest quartile, with an OR of 0.49 (95% CI: 0.41–0.59; *P* for trend < .0001). Subsequent analysis revealed a significant improvement in the threshold effect value to 4.28 for the potential relationship between NHHR and anemia. Subgroup analysis showed an inverse correlation in some subgroups. Alcohol consumption significantly affects the relationship between NHHR and anemia (interaction *P* < .05). Our study revealed that NHHR is inversely proportional to the prevalence of anemia in U.S. adults.

## 1. Introduction

About 1.8 billion people worldwide are affected by anemia, according to the GBDS 2019.^[1]^ Anemia is a significant public health challenge that affects people around the world. The occurrence of anemia is primarily driven by a combination of nutritional and non-nutritional factors, such as the lack of essential nutrients like iron, folic acid, or vitamin B12; decreased hematopoietic capacity; and by common infectious diseases, chronic illnesses, ongoing inflammation, or impaired immunity. Anemia is significantly associated with increased rates of morbidity, hospital admissions for any reason, and mortality among adults.^[2]^ Previous studies have identified anemia as an important prognostic marker for various severe conditions, including soft tissue sarcomas and heart failure.^[3,4]^ Apart from hemoglobin levels, what other biomarkers indicate anemia?

Several investigations have shown a correlation between blood lipid abnormalities and anemia.^[5–10]^ Low-density lipoprotein (LDL) receptor activity in anemic patients may be negatively correlated with plasma cholesterol levels.^[11]^ However, few studies have investigated the underlying mechanisms of this phenomenon. Altered absorption, abnormal synthesis, and changes in excretion, dilution, and redistribution are suggested mechanisms for altering serum cholesterol levels.^[11]^ Additionally, a rare condition known as hypertriglyceridemia thalassemia syndrome exists in clinical practice.^[12]^ Research indicates that lipid concentrations are often reduced in patients diagnosed with anemia compared to non-anemic individuals.^[13]^ Moreover, an increasing amount of research suggests that high-density lipoprotein cholesterol (HDL-C) may contribute to iron-induced damage to kidney tissues in patients with diabetes, which subsequently results in chronic anemia.^[14]^ Recent studies have shown that NHHR, newly identified as a risk indicator for atherosclerosis, can help to understand and predict the risks related to chronic kidney disease, metabolic syndrome, and nonalcoholic fatty liver disease (NAFLD).^[15–19]^

Nevertheless, the association between NHHR and anemia remains under-researched and indistinct. This research examines the relationship between NHHR and anemia prevalence in U.S. adults, based on data from the National Health and Nutrition Examination Survey (NHANES). NHHR’s role in anemic patients was scrutinized to reveal its significance.

## 2. Methods

### 2.1. Study population

NHANES is a comprehensive, ongoing evaluation of the US population’s nutrition and health status, utilizing intricate multi-stage probability sampling methods. For a detailed overview of NHANES’s continuous design, visit the CDC’s official website (https://www.cdc.gov/nchs/nhanes/index.htm). The National Center for Health Statistics (NCHS) ethical review board, upon approval of all study procedures, provided full disclosure and obtained consent from all participants.

**Figure 1.**
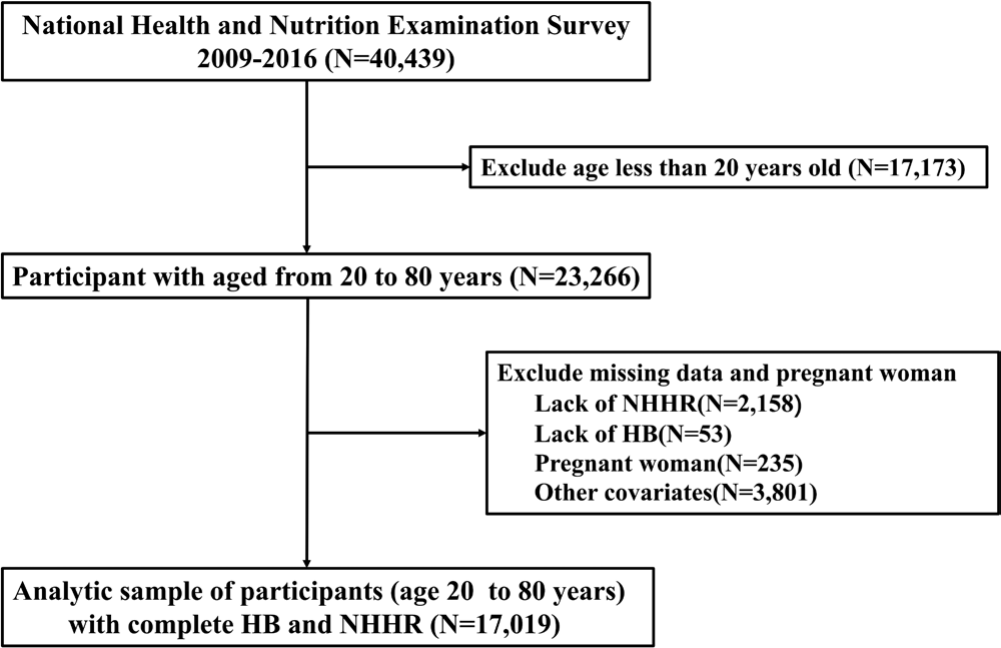
Flowchart for inclusion of participants in this study.

The current study analyzed data from the 4 NHANES cycles spanning from 2009–2010 to 2015–2016. We excluded 17,173 individuals under 20 years of age, 2158 individuals with missing NHHR data, 53 individuals lacking hemoglobin data, 235 pregnant individuals, and 3801 individuals without other covariates, resulting in a final cohort of 17,019 participants. The sample selection flowchart is shown in Figure 1.

### 2.2. NHHR and anemia

In this study, the NHHR exposure variable represents the ratio of non-HDL-C to HDL-C, which is a key cardiovascular risk factor. Non-HDL-C is calculated by subtracting HDL-C levels from total cholesterol (TC). According to WHO criteria, anemia at sea level is diagnosed when the hemoglobin level is <12 g/dL for women and <13 g/dL for men.

### 2.3. Covariates

The study considered gender, age, education level, marital status, race, and the familial income-to-poverty ratio (PIR). Additionally, it included assessments of alcohol intake and smoking. Biomarkers such as BMI (Body Mass Index), Aspartate Aminotransferase (AST), Alanine Aminotransferase (ALT), Lactate dehydrogenase (LDH), potassium, albumin, uric acid, serum iron, calcium, diabetes, creatinine, and hypertension were also analyzed. Marital status was categorized into “partnered” (including married and cohabitating individuals) and “single” (comprising never married, divorced, separated, and widowed). Income was measured using the US poverty threshold (PIR), with respondents classified as below poverty (PIR < 1.3), near poverty (1.3 ≤ PIR < 3.0), or not impoverished (PIR ≥ 3.0). Smoking intake was determined by participants’ self-reports of having smoked a minimum of 100 cigarettes during their lifetime. Alcohol intake was quantified based on whether participants reported consuming at least 12 alcoholic beverages annually. According to common guidelines, a BMI under 25 kg/m² is considered normal weight, while a BMI between 25 and 29.9 kg/m² is classified as overweight, and a BMI of 30 kg/m² or higher is considered obese. The presence of diabetes was confirmed if participants had 1 or more of the following: a self-report of diabetes, a fasting plasma glucose level of at least 126 mg/dL, a spontaneous blood glucose level of at least 200 mg/dL, a 2-hour postprandial glucose level of at least 200 mg/dL from an oral glucose tolerance test, a glycated hemoglobin (HbA1c) level of at least 6.5%, or if they were currently taking antidiabetic medication (including oral hypoglycemic agents or insulin). To establish a diagnosis of hypertension, a person must have self-reported hypertension or have a systolic blood pressure ≥ 140 mm Hg and/or a diastolic blood pressure ≥ 90 mm Hg, as determined by a healthcare professional. a systolic blood pressure of ≥ 140 mm Hg and/or a diastolic blood pressure of ≥90 mm Hg measured by a healthcare professional.

### 2.4. Statistical analysis

Empower Stats software (version 2.0) was utilized for statistical analysis. Baseline characteristics among the research subjects were delineated by anemia status (non-anemia and anemia groups). Continuous variables were summarized with means ± SD and analyzed using linear regression models. Categorical variables were encoded as proportions and subjected to chi-square tests for further analysis. NHHR and anemia were subjected to weighted multivariate logistic regression analysis to determine prevalence ORs and 95% CIs. Three multivariate models were constructed: Unadjusted model 1 was created, followed by adjustments for gender, age and race in model 2. Model 3, on the other hand, included all covariates. Threshold effect analysis was used to study the relationship and inflection point among NHHR and anemia. Nonlinearity between NHHR and anemia was examined by applying a smoothed curve fitting tool. Subgroup analyses were conducted to investigate the correlation between NHHR and anemia across various factors, such as gender, race, BMI, marital status, PIR, smoking and alcohol consumption, diabetes, and hypertension. Any *P* value lower than .05 was considered clinically significant and all analyses were conducted using the survey weights provided by NHANES.

## 3. Results

### 3.1. Baseline characteristics of participants

The research population comprised 17,019 participants, of whom 15,285 were non-anemic, with an average age of 48.73 ± 17.40 years (51.29% male, 48.71% female), and 1734 were anemic, with an average age of 55.50 ± 18.19 years (37.14% male, 62.86% female). The non-anemia group demonstrated a mean NHHR of 2.98 ± 1.47, compared to the anemia group’s 2.54 ± 1.24. This difference is statistically significant (*P* < .001). Statistical differences were observed between the 2 groups regarding the covariates of gender, age, education level, marital status, race, PIR, smoking status, alcohol consumption, BMI, Hemoglobin (HG), ALT, AST, LDH, potassium, uric acid, albumin, serum iron, calcium, creatinine, HDL-C, TC, diabetes, and hypertension (all *P* < .05) (Table 1).

**Table 1:** Baseline characteristics of participants by non-anemia and anemia groups.

Characteristics	Non-anemia (n = 15,285)	Anemia (n = 1734)	*P*-value
Age (yr)	48.73 ± 17.40	55.50 ± 18.19	<.001
Sex (%)			
Male	51.29%	37.14%	<.001
Female	48.71%	62.86%	
Race/ethnicity (%)			
Non-Hispanic White	45.49%	27.62%	<.001
Non-Hispanic Black	17.66%	40.89%	
Mexican American	14.63%	12.63%	
Other Hispanic	10.32%	8.71%	
Other Race	11.89%	10.15%	
Education level (%)			
<high school	22.02%	26.76%	<.001
high school	22.15%	23.59%	
>high school	55.83%	49.65%	
Marital status (%)			
Having a partner	59.63%	54.67%	<.001
Others	40.37%	45.33%	
PIR (%)			
<1.3	32.27%	38.35%	<.001
≥1.3, <3.0	30.29%	33.16%	
≥3.0	37.44%	28.49%	
Alcohol intake (%)			
Yes	73.87%	61.94%	<.001
No	26.13%	38.06%	
Smoking intake (%)			
Yes	45.36%	39.45%	<.001
No	54.64%	60.55%	
BMI category (%)			
Normal weight	28.54%	29.76%	<.001
Overweight	33.21%	28.55%	
Obese	38.25%	41.70%	
HG (g/dL)	14.33 ± 1.23	11.35 ± 1.09	<.001
ALT (U/L)	26.05 ± 21.50	20.27 ± 16.98	<.001
AST (U/L)	26.12 ± 17.34	24.79 ± 24.44	.004
LDH (U/L)	128.91 ± 30.17	134.78 ± 42.97	<.001
Potassium (mmol/L)	3.97 ± 0.33	4.02 ± 0.43	<.001
Uric acid (umol/L)	326.02 ± 82.99	322.91 ± 102.78	.149
Albumin (g/dL)	4.30 ± 0.32	4.04 ± 0.35	<.001
Serum iron (umol/L)	15.59 ± 6.06	10.29 ± 5.88	<.001
Calcium (mmol/L)	2.36 ± 0.09	2.31 ± 0.10	<.001
Creatinine (umol/L)	78.22 ± 25.87	100.78 ± 100.60	<.001
HDL-C (mmol/L)	1.36 ± 0.42	1.39 ± 0.43	.033
TC (mmol/L)	5.01 ± 1.06	4.57 ± 1.03	<.001
NHHR	2.98 ± 1.47	2.54 ± 1.24	<.001
Diabetes (%)			
Yes	17.70%	30.62%	<.001
No	82.30%	69.38%	
Hypertension (%)			
Yes	44.10%	59.80%	<.001
No	55.90%	40.20%	

Mean ± SD for continuous variables; the *P* value was calculated by the linear regression model (%) for categorical variables; the *P* value was calculated by the chi-square test.

Abbreviations: ALT = alanine aminotransferase, AST = aspartate aminotransferase, BMI = body mass index, HDL-C = high-density lipoprotein cholesterol, HG = hemoglobin, LDH = lactate dehydrogenase, NHHR = non-high-density lipoprotein cholesterol to high-density lipoprotein cholesterol ratio, PIR = ratio of family income to poverty, TC = total Cholesterol.

### 3.2. Associations between NHHR and anemia

The significant results demonstrated in Table 2 suggest that an elevated NHHR is positively associated with a diminished prevalence of anemia. The adjusted model showed a negative correlation between NHHR and anemia prevalence (OR = 0.82, 95% CI: 0.78–0.86). In other words, each 1 unit increase in NHHR was associated with a 18% decrease in anemia prevalence. After categorizing NHHR into quartiles, the association with anemia remained significant. Table 2 shows that participants in the highest NHHR quartile had a significantly lower prevalence of anemia, with a 51% decrease (OR = 0.49, 95% CI: 0.41–0.59; *P* for trend < .0001) compared to those in the lowest quartile.

**Table 2:** The odds ratio for the relationship between non-high-density lipoprotein cholesterol to high-density lipoprotein cholesterol ratio and anemia.

Exposure	Model 1		Model 2		Model 3	
	OR (95% CI) *P*-value		OR (95% CI) *P*-value		OR (95% CI) *P*-value	
NHHR (continuous)	0.77 (0.74, 0.80)	<.0001	0.83 (0.80, 0.87)	<.0001	0.82 (0.78, 0.86)	<.0001
NHHR (quartile)						
Quartile 1	Reference		Reference		Reference	
Quartile 2	0.78 (0.69, 0.89)	.0002	0.82 (0.72, 0.94)	.0037	0.77 (0.67, 0.90)	.0007
Quartile 3	0.60 (0.52, 0.68)	<.0001	0.67 (0.58, 0.77)	<.0001	0.60 (0.51, 0.70)	<.0001
Quartile 4	0.40 (0.34, 0.47)	<.0001	0.52 (0.44, 0.61)	<.0001	0.49 (0.41, 0.59)	<.0001
*P* for trend	<.0001		<.0001		<.0001	

Model 1: no covariates were adjusted. Model 2:age, gender and race were adjusted. Model 3: age, gender, race, education level, marital status, PIR, alcohol intake, smoking intake, BMI, ALT, AST, LDH, Potassium, Uric acid, albumin, serum iron, calcium, creatinine, diabetes and hypertension were adjusted.

Abbreviations: ALT = alanine aminotransferase, AST = aspartate aminotransferase, BMI = body mass index, LDH = lactate dehydrogenase, NHHR = non-high-density lipoprotein cholesterol to high-density lipoprotein cholesterol ratio, PIR = ratio of family income to poverty.

### 3.3. The nonlinearity and threshold effect of NHHR and anemia were analyzed

**Figure 2.**
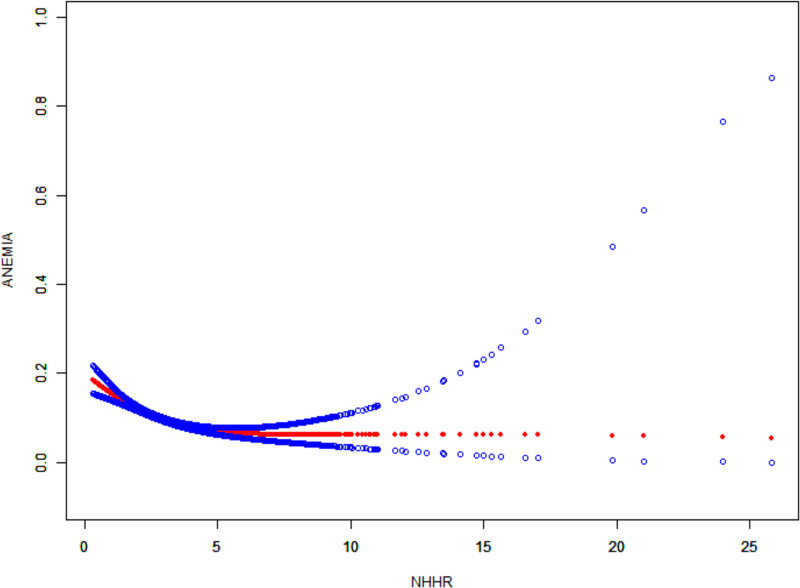
The nonlinear associations between non-high-density lipoprotein cholesterol to high-density lipoprotein cholesterol ratio and anemia. The solid red line illustrates the smooth curve fitting. Blue bands indicate the 95% confidence interval of the fit.

Smoothed curve fitting showed that NHHR was negatively correlated with anemia prevalence (Fig. 2). Analysis revealed a threshold effect value of 4.28 for the relationship between NHHR and anemia among all participants. In Table 3, an NHHR level of 4.28 was found to be critical. As NHHR falls below this value, anemia prevalence sharply reduces by 15% for every unit increase. The associated effect size is 0.75, indicating that with a 1 unit increase in NHHR, the OR for anemia prevalence is around 0.75 (95% CI: 0.71–0.81). On the contrary, there was no appreciable change in the prevalence of anemia when the NHPA was above 4.28. An inflection point for NHHR’s impact on anemia were established through smoothed curve fitting and threshold effect analysis.

**Table 3:** Threshold effect analysis of non-high-density lipoprotein cholesterol to high-density lipoprotein cholesterol ratio in anemia.

Anemia	Adjusted OR (95% CI), *P*-value
Fitting by the standard linear model	0.82 (0.78, 0.86), <.0001
Fitting by the 2-piecewise linear model	
Saturation point	4.28
NHHR < 4.28	0.75 (0.71, 0.81), <.0001
NHHR > 4.28	1.02 (0.92, 1.14), .6945
*P* for Log-likelihood ratio	<.001

Age, gender, race, education level, marital status, PIR, alcohol intake, smoking intake, BMI, ALT, AST, LDH, potassium, Uric acid, albumin, serum iron, calcium, creatinine, diabetes and hypertension were adjusted.

Abbreviations: ALT = alanine aminotransferase, AST = aspartate aminotransferase, BMI = body mass index, LDH = lactate dehydrogenase, NHHR = non-high-density lipoprotein cholesterol to high-density lipoprotein cholesterol ratio, PIR = ratio of family income to poverty.

### 3.4. Subgroup analysis

**Figure 3.**
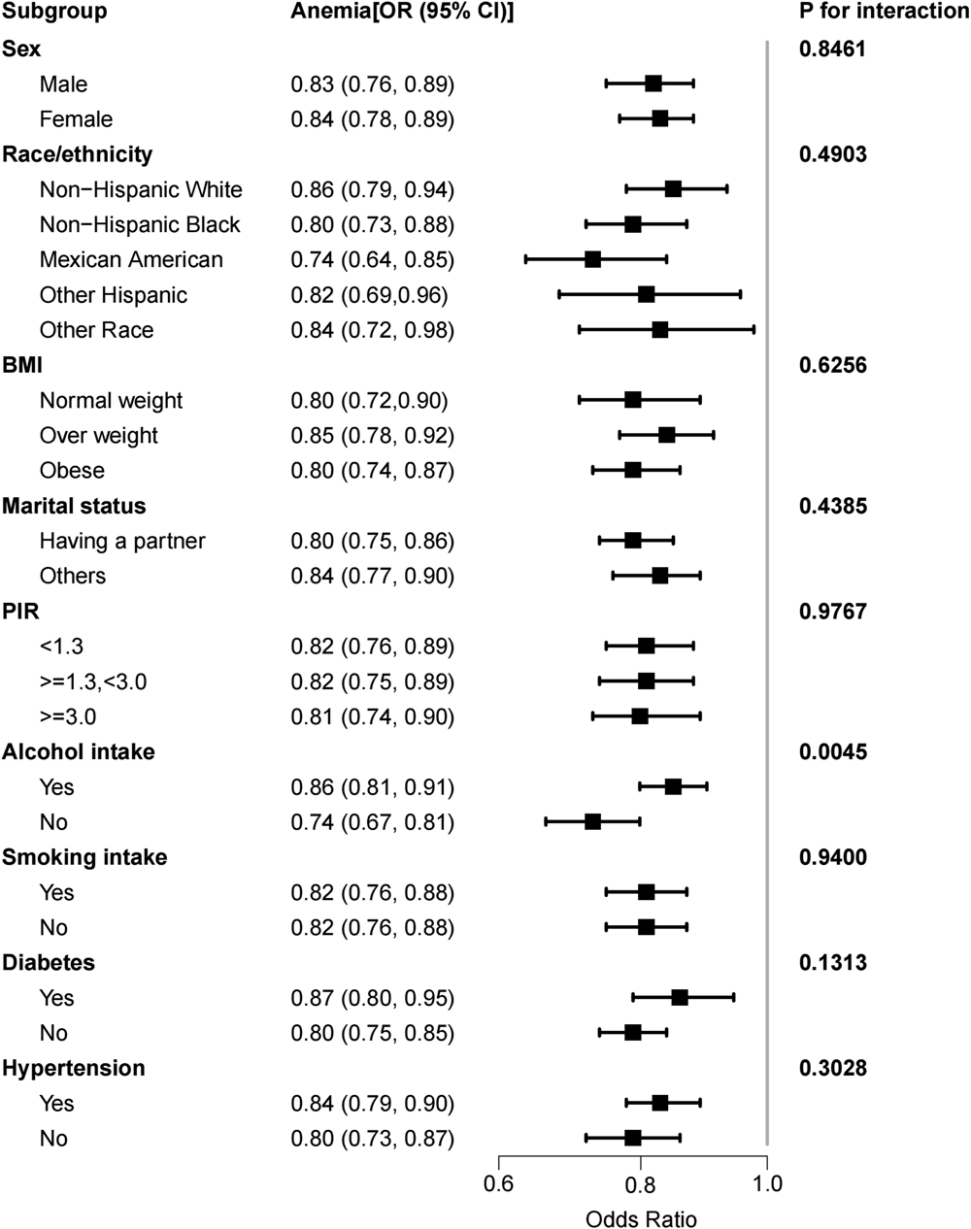
Subgroup analysis of the association between non-high-density lipoprotein cholesterol to high-density lipoprotein cholesterol ratio and anemia. Age, gender, race, education level, marital status, PIR, alcohol intake, smoking intake, BMI, ALT, AST, LDH, potassium, uric acid, albumin, serum iron, calcium, creatinine, diabetes and hypertension were adjusted. ALT = alanine aminotransferase, AST = aspartate aminotransferase, BMI = body mass index, LDH = lactate dehydrogenase, PIR = ratio of family income to poverty.

Current findings reveal inconsistent associations between NHHR levels and anemia. NHHR was found to be significantly correlated with the analyzed data. and anemia across various demographics and health statuses: gender, race, BMI, marital status, PIR categories (<1.3; ≥1.3, <3.0; ≥3.0), smokers and nonsmokers, non-diabetes or diabetes subjects and participants with or without hypertension. Furthermore, interaction analyses indicated significant differences in the relationship between NHHR and anemia based on alcohol consumption, with it significantly influencing this association (interaction *P* < .05) (Fig. 3; Table S1, Supplemental Digital Content, http://links.lww.com/MD/O210).

## 4. Discussion

In this cross-sectional analysis involving 17,019 individuals, researchers noted a reverse correlation between the NHHR and anemia prevalence, suggesting that lower NHHR could be a factor in the heightened occurrence of anemia. Concurrently, the study unveiled a threshold effect value of 4.28 for the NHHR when assessing its connection with anemia. Below this threshold, there was a negative correlation between NHHR and anemia prevalence. However, after reaching this inflection point, the correlation became statistically insignificant. Subgroup analysis revealed a significant influence of alcohol consumption on this association. Research indicates that alcohol consumption, especially frequent consumption, is linked to a high prevalence of dyslipidemia.^[20]^Additionally, it is well known that alcohol consumption directly impairs liver function,^[21]^ which in turn affects lipid metabolism, exacerbating dyslipidemia^[22,23]^ Therefore, our research confirms that alcohol consumption can directly affect the connection between NHHR and anemia.

This study has initiated a preliminary examination into the link between NHHR and anemia, as well as the recognition of increasing evidence on NHHR as a precise indicator for the risk of lipid-related diseases.^[17,24]^ Despite the relatively small number of direct studies, a wealth of evidence demonstrates an association between anemia and a range of lipid-related factors.^[5,11,25–27]^ Additionally, research has demonstrated a significant correlation between obesity and anemia.^[28]^ Animal research indicates that a high-fat diet results in lipid disorders that precipitate obesity. This condition correlates with leukocytosis, largely attributable to neutrophilia. Enhanced G-CSF production in the bone marrow of obese animals could explain the rise in leukocyte numbers. Moreover, researchers found that rats on a high-fat diet exhibited a significant increase in the release of interleukins (IL-1 and IL-6), a protein called nuclear factor kappa B, and tumor necrosis factor alpha from mesenchymal stem cells. These cytokines, by hindering MSC differentiation into adipocytes, may prompt bone marrow to favor osteoblast differentiation. Altered bone marrow microenvironments are likely responsible for disrupted hematopoietic cell development.^[29]^ Pronounced obesity in humans often leads to red blood cell production disorders, such as anemia and iron metabolism imbalances. Elevated IL-6 and leptin levels explain these erythropoietic disturbances. Obesity-related inflammation prompts an elevated release of hepatic phospholipids from both the liver and adipose tissues. Hepcidin limits erythropoiesis by inhibiting iron absorption in intestinal cells and sequestration in macrophages.^[28,30]^ Dyslipidemia from a high-fat diet contributes to obesity, which is associated with increased leukocytes and inflammation. This, in turn, stimulates enhanced hepcidin release, interfering with iron absorption.^[28]^ Metabolic syndrome, a condition characterized by insulin resistance, is frequently associated with dyslipidemia. Elevated insulin resistance can lead to persistent low-grade inflammation through the upregulation of pro-inflammatory cytokines, which can negatively impact anemia.^[6,31–33]^ NHHR, which combines CDL-C and HDL-C, may theoretically serve as a robust marker for evaluating anemia. To our current understanding, this research marks the first time a correlation between NHHR and anemia has been examined. NHHR is considered a novel lipid marker for atherosclerosis, an individual risk factor of atherosclerotic plaques, but also an important biomarker for the prevention of plaque formation.^[34]^ Its diagnostic value in predicting insulin resistance, metabolic syndrome and NAFLD has surpassed that of traditional lipid markers.^[16,18,35]^ The NHHR’s robust association with a variety of diseases confirms its efficacy in lipid management. This investigation found a potentially negative association between the NHHR and the anemia incidence, highlighting the NHHR as a useful tool for understanding the link between lipid metabolism and anemia.

This study has the following advantages. Firstly, it utilizes data from NHANES, which consists of nationally representative, population-based samples collected through a standardized protocol, characterized by a large sample size. Additionally, the authors adjusted for confounding variables, enhancing the reliability of the results. Subgroup analyses further examined the robustness of NHHR and anemia association across diverse populations. However, the study’s limitations warrant attention. The creation of determining causality was ruled out by the cross-sectional design. Furthermore, despite considering numerous variables, the potential influence of unaccounted confounding factors remains.

## 5. Conclusion

Our research shows a negative relationship between NHHR and anemia prevalence, suggesting that higher NHHR levels are associated with a diminished prevalence of anemia. These findings highlight NHHR’s importance in managing anemia. However, validation of these findings necessitates additional large-scale prospective studies.

## Acknowledgments

We appreciate the National Health and Nutrition Examination Survey for supplying the data and acknowledge the selfless dedication of all participants.

## Author contributions

**Conceptualization:** Nengneng Cao, Jiajia Wang.

**Data curation:** Nengneng Cao, Jiajia Wang, Xunyi Jiao.

**Formal analysis:** Nengneng Cao, Jiajia Wang, Xunyi Jiao, Furun An.

**Funding acquisition:** Furun An, Zhimin Zhai.

**Investigation:** Jinli Zhu, Xunyi Jiao.

**Methodology:** Nengneng Cao, Jiajia Wang, Jinli Zhu, Xunyi Jiao.

**Project administration:** Zhimin Zhai.

**Software:** Nengneng Cao, Jiajia Wang, Jinli Zhu, Xunyi Jiao.

**Supervision:** Furun An.

**Validation:** Nengneng Cao.

**Visualization:** Nengneng Cao, Jiajia Wang.

**Writing – original draft:** Nengneng Cao, Jiajia Wang, Jinli Zhu, Xunyi Jiao.

**Writing – review & editing:** Furun An, Zhimin Zhai.

## Supplementary Material


