# Supplementary material for: The relationship between non-HDL cholesterol to HDL cholesterol ratio (NHHR) and anemia: A cross-sectional study of NHANES, 2009 to 2016
Source: Medicine (Baltimore). 2024 Dec 13;103(50):e40976. doi: 10.1097/MD.0000000000040976 (PMC11651433; doi:10.1097/MD.0000000000040976)
Supplement: Supplementary file 1 [file medi-103-e40976-s001.docx]

**Supplemental Table 1** Subgroup analysis of the association between non-high-density lipoprotein cholesterol to high-density lipoprotein cholesterol ratio and Anemia

| Subgroup | Anemia [OR (95% CI)] | P for interaction |
| --- | --- | --- |
| Sex  Male  Female | 0.83 (0.76, 0.89)  0.84 (0.78, 0.89) | .8461 |
| Race/ethnicity  Non-Hispanic White  Non-Hispanic Black  Mexican American  Other Hispanic  Other Race | 0.86 (0.79, 0.94)  0.80 (0.73, 0.88)  0.74 (0.64, 0.85)  0.82 (0.69, 0.96)  0.84 (0.72, 0.98) | .4903 |
| BMI  Normal weight  Overweight  Obese | 0.80 (0.72, 0.90)  0.85 (0.78, 0.92)  0.80 (0.74, 0.87) | .6256 |
| Marital status  Having a partner  Others | 0.80 (0.75, 0.86)  0.84 (0.77, 0.90) | .4385 |
| PIR  <1.3  ≥1.3, <3.0  ≥3.0 | 0.82 (0.76, 0.89)  0.82 (0.75, 0.89)  0.81 (0.74, 0.90) | .9767 |
| Alcohol intake  Yes  No | 0.86 (0.81, 0.91)  0.74 (0.67, 0.81) | .0045 |
| Smoking intake  Yes  No | 0.82 (0.76, 0.88)  0.82 (0.76, 0.88) | .9400 |
| Diabetes  Yes  No | 0.87 (0.80, 0.95)  0.80 (0.75, 0.85) | .1313 |
| Hypertension  Yes  No | 0.84 (0.79, 0.90)  0.80 (0.73, 0.87) | .3028 |

Age, gender, race, education level, marital status, PIR, alcohol intake, smoking intake, BMI, ALT, AST, LDH, potassium, Uric acid, albumin, serum iron, calcium, creatinine, diabetes and hypertension were adjusted.

Abbreviation: PIR=Ratio of family income to poverty, BMI=Body mass index, ALT=Alanine Aminotransferase, AST=Aspartate Aminotransferase, LDH=Lactate dehydrogenase, NHHR=non-high-density lipoprotein cholesterol to high-density lipoprotein cholesterol ratio.
